# Transcranial Magnetic Stimulation of Human Adult Stem Cells in the Mammalian Brain

**DOI:** 10.3389/fncir.2016.00017

**Published:** 2016-03-17

**Authors:** Karlea L. Kremer, Ashleigh E. Smith, Lauren Sandeman, Joshua M. Inglis, Michael C. Ridding, Simon A. Koblar

**Affiliations:** ^1^School of Paediatrics and Reproductive Health, The Robinson Research Institute, The University of AdelaideAdelaide, SA, Australia; ^2^School of Medicine, The Stroke Research Programme, The University of AdelaideAdelaide, SA, Australia; ^3^Alliance for Research in Exercise Nutrition and Activity (ARENA), School of Health Science, Sansom Institute for Health Research, University of South AustraliaAdelaide, SA, Australia

**Keywords:** stroke, dental pulp stem cells, transcranial magnetic stimulation, glutamate, rat

## Abstract

**Introduction**: The burden of stroke on the community is growing, and therefore, so is the need for a therapy to overcome the disability following stroke. Cellular-based therapies are being actively investigated at a pre-clinical and clinical level. Studies have reported the beneficial effects of exogenous stem cell implantation, however, these benefits are also associated with limited survival of implanted stem cells. This exploratory study investigated the use of transcranial magnetic stimulation (TMS) as a complementary therapy to increase stem cell survival following implantation of human dental pulp stem cells (DPSC) in the rodent cortex.

**Methods**: Sprague-Dawley rats were anesthetized and injected with 6 × 10^5^ DPSC or control media via an intracranial injection, and then received real TMS (TMS_0.2 Hz_) or sham TMS (TMS_sham_) every 2nd day beginning on day 3 post DPSC injection for 2 weeks. Brain sections were analyzed for the survival, migration and differentiation characteristics of the implanted cells.

**Results**: In animals treated with DPSC and TMS_0.2 Hz_ there were significantly less implanted DPSC and those that survived remained in the original cerebral hemisphere compared to animals that received TMS_sham_. The surviving implanted DPSC in TMS_0.2 Hz_ were also found to express the apoptotic marker Caspase-3.

**Conclusions**: We suggest that TMS at this intensity may cause an increase in glutamate levels, which promotes an unfavorable environment for stem cell implantation, proliferation and differentiation. It should be noted that only one paradigm of TMS was tested as this was conducted as a exploratory study, and further TMS paradigms should be investigated in the future.

## Introduction

Stroke is a leading cause of long-term severe disability (Truelsen et al., 2007), resulting in persistent neurological deficits and profound physical deconditioning that propagates disability and worsens cardiovascular risk (Ivey et al., 2006). Indeed, nearly half of all older stroke survivors experience ongoing moderate to severe disability. Consequently, there is a pressing need for the development of novel and effective therapies. One of the most promising approaches is exogenous stem cell therapy, which involves the implantation of a stem cell source from outside of the brain. Human dental pulp stem cells (DPSC) are an attractive option for exogenous stem cell therapy (Leong et al., 2012) and have many advantages as a potential cell based therapy for stroke. Firstly, teeth provide a clinically accessible source of human adult stem cells. Secondly, human DPSC when exposed to the appropriate environment have neurogenic potential, generate electrophysiologically functional neurons and can influence a host’s nervous system to alter connectivity (Arthur et al., 2008). Thirdly, *ex vivo* human DPSC have a high proliferative capacity that can generate sufficient cells for future human stem cell therapy (Gronthos et al., 2000) and fourthly, due to improved dental hygiene, many older adults possess their own teeth which could then be used to generate their own human DPSC for autologous transplantation. The mechanism of action of cell-based therapies including human DPSC transplantation, is likely through a variety of actions including direct cell replacement, immunomodulation, neuroprotection, angiogenesis and neuroplasticity (Leong et al., 2012).

There are currently many obstacles to overcome when using exogenous stem cell therapy. One such problem is the limited survival, differentiation and connectivity of DPSC implants. It has been reported that as few as 2.3% of implanted human DPSC survived following intracerebral transplantation of human DPSC into the rodent brain (Leong et al., 2012). Despite the low survival rate of implanted DPSC, significant functional improvement was seen in animals that received the DPSC compared to vehicle only controls (Leong et al., 2012). This highlights the exciting potential for stem cell therapy to improve functional outcomes following stroke. However, a combination of therapies which promote stem cell survival, differentiation and the development of appropriate connectivity between implanted cells and the local neuronal population may be necessary to provide the optimum outcome. Mounting evidence indicates that a major cause of limited survival of transplanted cells is due to a lack of sufficient growth factors at the transplant site (Wang et al., 2013). The deficiency in trophic support in the post stroke brain is the result of the pathophysiological cascade following an occluded blood vessel and disrupted cerebral blood flow. This cascade generates a number of events, including increased apoptosis due to elevated Ca^2+^, impaired mitochondrial function, energy depletion and excitotoxicity caused by the uncontrolled release of glutamate, all making the brain a hostile environment (Durukan and Tatlisumak, 2007). These factors not only prevent proliferation and survival of implanted stem cells, but also the surviving endogenous cells, as regeneration of endogenous neurons in the infarct area is minimal (Wang et al., 2013).

Transcranial Magnetic Stimulation (TMS) is one technique that may be useful in optimizing conditions for stem cell survival. TMS was developed as a non-invasive and well tolerated method for the focal stimulation of human cortical areas and has been used in both research and clinical settings (Rothwell, 1997). There is indirect evidence from both human and animal studies that have indicated that TMS can provide an environment which may prove beneficial for stem cell survival. Recent studies have also demonstrated that TMS increases endogenous neurotrophins in rodents brains such as glutamate and brain-derived neurotrophic factor (BDNF) which are both known to induce signaling pathways that lead to neurogenesis (Ikonomidou and Turski, 2002; Ma et al., 2013; Tan et al., 2013). Low frequency magnetic stimulation has been proposed to increase neurotrophic factors BDNF and neurotrophic growth factor (NGF; Tan et al., 2013) which are factors known to be beneficial for stem cell growth and survivial (Bates and Rodger, 2015). As exogenous neurotrophins are unable to penetrate the blood brain barrier, TMS application is a feasible, cost effective and well tolerated method to non-invasively increase neurotrophin content in the brain, eliminating the risk of increasing intracranial pressure and traumatic brain injury if these factors were to be intracerebrally injected (Tan et al., 2013). TMS has also recently been translated from humans to a variety of animal models including rats and cats to examine cortical changes in animal disease models in order to investigate the underlying physiology of common TMS phenomena (Funke and Benali, 2010, 2011).

Therefore the aim of this exploratory pilot study is to identify a novel approach using a combination of TMS therapy following stem cell injection in the mammalian brain that will optimize conditions within the brain for stem cell survival and the development of appropriate connectivity. We hypothesize that repeated periods of TMS following DPSC transplantation will produce changes within the cortex that are beneficial for stem cell survival and differentiation.

## Materials and Methods

### Animal and Experimental Preparation

Experiments were carried out on Sprague-Dawley rats, male, weighing 300 g (University of Adelaide, Laboratory Animal Services). A total of 26 rats were used in this study. The animals were maintained on a 12 h light/dark cycle with *ad libitum* access to food and water. All experimental procedures complied with the Australian Code of Practice for the Care and Use of Animals for Scientific Purposes and were used with permission from the University of Adelaide Animal Ethics Committee (approval number M-2011-108).

### Dental Pulp Stem Cell Culture and Preparation

Human DPSC were previously isolated from adult impacted third molars by digestion of pulp tissue (Arthur et al., 2008) and characterized (Gronthos et al., 2002) and retrovirally transduced to express green fluorescent protein (GFP; Arthur et al., 2009). Human Ethics for DPSC isolation was obtained from The University of Adelaide H-73–2003. DPSC at passages 5–7 were cryopreserved in 10% dimethyl sulfoxide in fetal calf serum. Cells were thawed and passaged at least one time prior to use for intracerebral transplantation. DPSC were cultured as previously described , in α-modified Eagle’s medium (Sigma-Aldrich, St. Louis, MO, USA) supplemented with 10% fetal calf serum, 100 μM L-ascorbic acid 2-phosphate (Wako, Richmond, VA, USA), 2 mM L-glutamine, 100 U/mL penicillin and 100 μg/mL streptomycin at 37°C in 5% CO_2_. Cells were dissociated with trypsin and resuspended in α-modified Eagle’s medium without supplements at a concentration of 1.5 × 10^5^ cells/μL. Cell viability was determined using the trypan blue exclusion method.

### Dental Pulp Stem Cell Injection

Prior to surgery all animals were housed in an animal care facility for at least a week. Animals were anesthetized with isoflurane and 1.5 L/min oxygen (3% induction, 1.5–2% maintenance). Animals were randomly selected to receive human DPSC (6 × 10^5^ cells in 4 μL total volume) or a media only injection. The injection of cell suspension or media was made into the right hemisphere parenchyma at two stereotaxic coordinates (anterior-posterior, medial-lateral relative to bregma; dorsal-ventral from dura): 2 μL into the striatum (−0.40, +4.00; −5.50 mm) and another 2 μL into the cortex (−1.40, +4.00, −1.75 mm) using a stereotaxic frame (Kopf instruments, Tujunga, CA, USA).

### Transcranial Magnetic Stimulation of the Mammalian Brain

**Figure 1:**
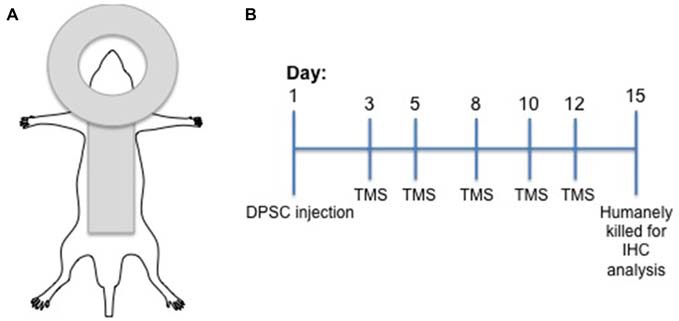
**Experimental design. (A)** Diagramatic representation of the transcranial magnetic stimulation (TMS) coil position in relation to rat position. **(B)** Experimental timeline including dental pulp stem cells (DPSC) injection and TMS treatments.

Following surgery animals recovered for 48 h and then received TMS on days 3, 5, 8, 10 and 12 post surgery (Figure 1). Animals injected with DPSC were randomized to receive real stimulation (TMS_0.2 Hz_) or sham TMS stimulation (TMS_Sham_). Animals injected with control media received TMS_0.2 Hz_. During TMS application animals were anesthetized using a mixture of Xylazil (4 mg/kg) and Ketamine (30 mg/kg). TMS_0.2 Hz_ was applied with a round coil (external diameter 13 cm; Magstim 200 (Magstim Co., Dyfed, UK)) connected to a monophasic stimulator with the center of the coil placed over the vertex with the handle pointing posteriorly. The coil was placed so that the coil current flowed in a clockwise direction so the right hemisphere was preferentially stimulated (see Figure 1). TMS was administered using 60% maximal stimulator output for 15 min at 0.2 Hz. This was the maximum intensity that could be applied at 0.2 Hz for 15 min without the coil overheating (determined in pilot studies). This intensity of resulted in a motor response in all the anesthetized animals (hind limb motor response was seen in all animals). Sham stimulation was administered by tilting the coil 90°. There was no direct stimulation of the rat brain during sham stimulation.

### Immunohistochemistry and Imaging

Two days following the final post-surgical TMS session rats were deeply anesthetized (5% isoflurane) and transcardially perfused with ice-cold saline followed by 4% (w/v) paraformaldehyde in phosphate-buffered saline. Brains were removed and post-fixed in the same fixative at 4°C overnight. Following fixation, brains were cryoprotected with 30% sucrose and embedded in optimum cutting temperature compound. Serial coronal sections of 8–12 μm were taken through the brain tissue surrounding the injection site.

**Figure 2:**
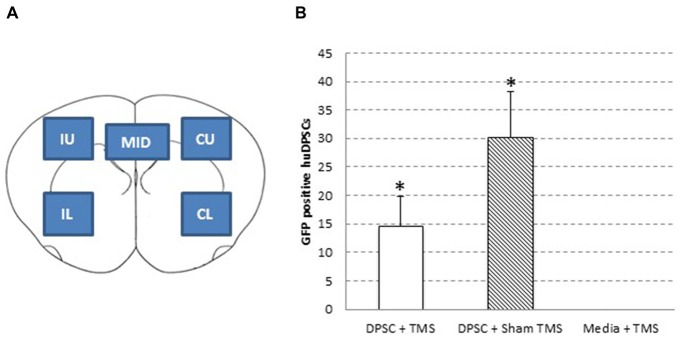
**Human dental pulp stem cell (huDPSC) numbers are decreased in animals that received TMS compared to those that received sham TMS. (A)** Schematic outline of the five areas used to count huDPSCs in rat brain sections: IU, ipsilateral upper; IL, ipsilateral lower; CU, contralateral upper; CL, contralateral lower; and MID, midline. **(B)** The average number of huDPSCs counted in five areas of 8 μm rat brain sections. *n* = 4–13 ± SEM, *ANOVA *p* = 0.0572, *t*-test, *p* = 0.0469.

Double immunohistochemistry studies were performed by blocking sections with 10% normal donkey serum in phosphate-buffered saline containing 0.3% Triton X-100, before DPSC were identified using goat anti-GFP (1:250; Rockland, Gilbertsville, PA, USA^1^). An antibody against Nestin was used for identification of neural stem cells (mouse anti-nestin; 1:100; Abcam, Cambridge, U.K.^2^), and cell death via Caspase-3 pathway was investigated with Cleaved Caspase-3 (1:1600; Cell Signaling Technology^3^). Other primary antibodies used were mouse anti- neuronal nuclei (NeuN) for mature neurons (1:500, Millipore), rabbit anti-glial fibrillary acidic protein (GFAP) to stain for astrocytes (1:250, DAKO, Glostrup, Denmark) and mouse anti-β-III Tubulin (1:500, Millipore). Secondary antibodies were either alexa fluor 488-conjugated anti-goat IgG, 568-conjugated anti-rabbit IgG or 647-conjugated anti-mouse IgG (1:200, Molecular Probes, Invitrogen Carlsbad, CA, USA^4^). Antibodies were diluted in blocking solution. All sections were mounted with ProLong Gold Antifade with DAPI reagent (Invitrogen, Carlsbad, CA, USA^4^). No primary antibody and no secondary antibody controls were used in triplicate to ensure accuracy of staining. Images were taken using Zeiss Axioscope microscope with Axiocam MRm high-resolution camera and Axiovision 4.8.1 imaging Software. DPSC numbers were determined by counting the numbers of GFP positive cells in five brain areas (Figure 2A) in up to 13 serial brain sections taken from four different rats in each treatment group.

### *In Vitro* Glutamate Activity Assay

Human DPSC were plated at a density of 5 × 10^4^ cells / well in 100 μL of DPSC cell culture medium in a 96 well plate. Cells were allowed to adhere for 3 h under standard culture conditions. At 3 h post-plating, cells were incubated with various concentrations of Glutamate (Sigma, G1251) for either 15 min to simulate the up-regulation of glutamate seen during TMS, 24 h or one trial where glutamate was added for 15 min, followed by a washout step for 45 min with the addition of glutamate and washout was repeated for a total of six times to replicate the *in vivo* paradigm of TMS used with each experiment completed in triplicate. Twenty-four hours after cells were initially plated, 10 μL of WST-1 cell proliferation reagent (Scientifix, Cheltenham, VIC, Australia) was added and cell proliferation was read at 450 nm and 690 nm.

### Statistics

GraphPad Prism (version 6.01) Software was used to perform a student’s *t*-test or one-way ANOVA, as appropriate.

## Results

### Number of DPSC were Decreased in TMS Treated Rat Brains

Five areas of the brain were analyzed and counted for GFP expressing DPSC (Figure 2A). This part of the study compared the total number of GFP expressing DPSC from all five sections in animals treated with real TMS_0.2 Hz_ or TMS_sham_. The findings from analysis of GFP expressing DPSC in brain sections showed animals that received an injection of DPSC followed by TMS had significantly less surviving DPSC compared to animals that received an injection of DPSC but received sham stimulation, *p* = 0.0469 *t*-test (Figure 2B).

### Decreased Trans-hemispheric Migration of Implanted DPSC in TMS Treated Brains

**Figure 3:**
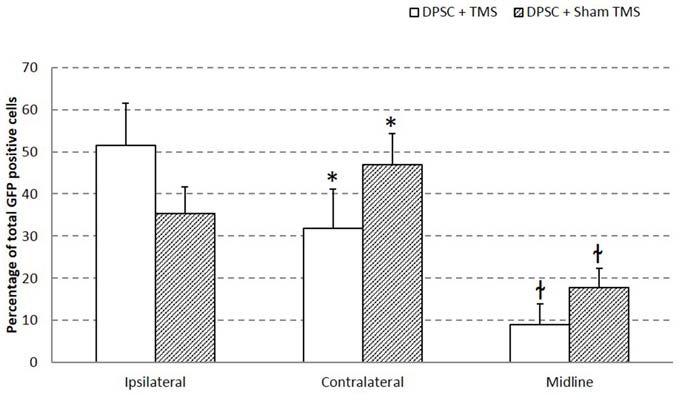
**Migration of implanted huDPSCs is decreased by TMS.** Brain sections of rats treated with DPSCs + TMS show less migration from the side of injections (ipsilateral hemisphere) to the contralateral hemisphere. Data is expressed as mean ± SEM, *p = 0.0458 *t*-test, ^ł^*p* = 0.0229 *t*-test.

Migration of the implanted DPSC from their injection site across the brain hemispheres was investigated. GFP expressing DPSC were counted in five areas as shown in Figure 2A. Ipsilateral (indicating the hemisphere of injection) upper and lower, contralateral upper and lower and a position in the midline including the corpus collosum. Migration of DPSC was seen in animals that received TMS_sham_ from the site of injection to the contralateral hemisphere. Half of the surviving DPSC from the TMS_0.2 Hz_ group were found in the same hemisphere of implantation. Animals that received TMS_sham_ were seen to have a lower percentage of implanted DPSC in the hemisphere of implantation compared to the contralateral side and the midline. It appeared that TMS_0.2 Hz_ significantly inhibited migration of the DPSC to the contralateral hemisphere, *p* = 0.0458 (*t*-test; Figure 3).

### Implanted DPSC Co-localize with Neural Stem Cell Marker Nestin

**Figure 4:**
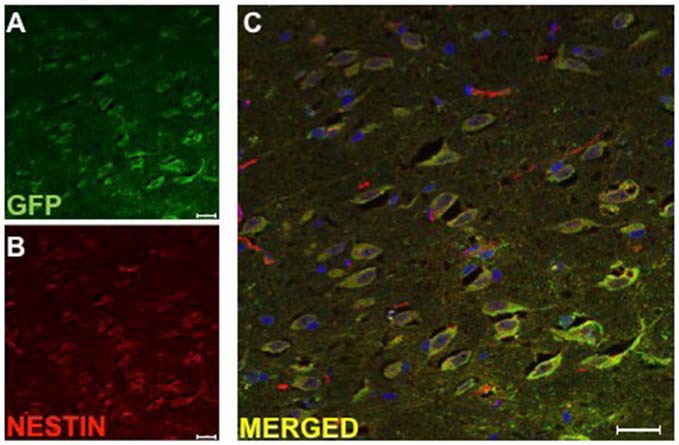
**Implanted huDPSCs co-localize with neural stem cell marker nestin in animals treated with TMS_(0.2 Hz)_.** TMS treated brain sections stained for **(A)** Green fluorescent protein (GFP) expressing huDPSCs (Green) and **(B)** Neural stem cell marker nestin (Red). **(C)** Merged image shows all GFP expressing huDPSCs co-localize with nestin. Scale bar represents 25 μm in each panel.

The surviving implanted DPSC were assessed for their differentiation potential *in vivo* using immunohistochemistry. Co-localization of GFP and antibodies against βIII-tubulin, GFAP and nestin were used to identify implanted DPSC that had differentiated into neurons, astrocytes, or had maintained a progenitor-like phenotype, respectively. In our previous study, GFP transduced human DPSC implanted into the rodent brain following stroke (Leong et al., 2012) expressed immunocytochemical markers consistent with differentiation into astrocytes and neurons. In contrast, we found no co-localization of GFP implanted human DPSC with βIII-tubulin, mature neuronal marker NeuN or astrocytic marker GFAP in eight brains that were examined (data not shown). Instead, all surviving DPSC in the TMS_0.2 Hz_ treated group co-localized with nestin, a neural stem cell marker. This result would suggest that less mature DPSC survived TMS_0.2 Hz_ treatment and that TMS_0.2 Hz_ selected for a progenitor-like cell from the total DPSC population that was implanted (Figure 4).

### Implanted DPSC Express Cell Death Marker Caspase-3

**Figure 5:**
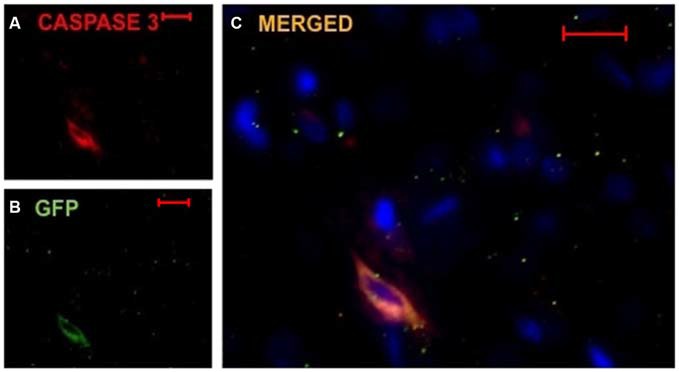
**Implanted huDPSCs co-localize with cell death marker Caspase-3 in animals treated with TMS_(0.2 Hz)_.** TMS treated brain sections stained for DAPI (blue) **(A)** Caspase-3 expressing cells (red) and **(B)** GFP expressing implanted huDPSCs (green). **(C)** Merged image shows GFP expressing huDPSCs co-localize with Caspase-3. Scale bar represents 20 μm in each panel.

Due to the decreased number of implanted DPSC found in TMS_0.2 Hz_ treated animals, we investigated whether the surviving cells expressed an apoptotic marker and destined for cell death. Caspase-3 is one of the key executioners of apoptosis as it is either partially or totally responsible for the proteolytic cleavage of many key proteins (Fernandes-Alnemri et al., 1994). In animals which had DPSC implanted and TMS_0.2 Hz_, implanted cells (between 80–90% of DPSC) were seen to co-localize with the Caspase-3 marker (Figure 5). This suggests that TMS or a by-product following TMS caused activation of the Caspase-3 apoptotic pathway that resulted in reduced survival of implanted DPSC. Caspase-3 staining was carried out on both TMS_0.2 Hz_ treated and TMS_sham_ animals, with TMS_sham_ animals showing no Caspase-3 co-localization in implanted human DPSC, and no Caspase-3 expression in the sub ventricular zone (data not shown). This suggests Caspase-3 expression was localized to the implanted human DPSC population.

### Up-Regulation of Glutamate Decreases DPSC Proliferation

**Figure 6:**
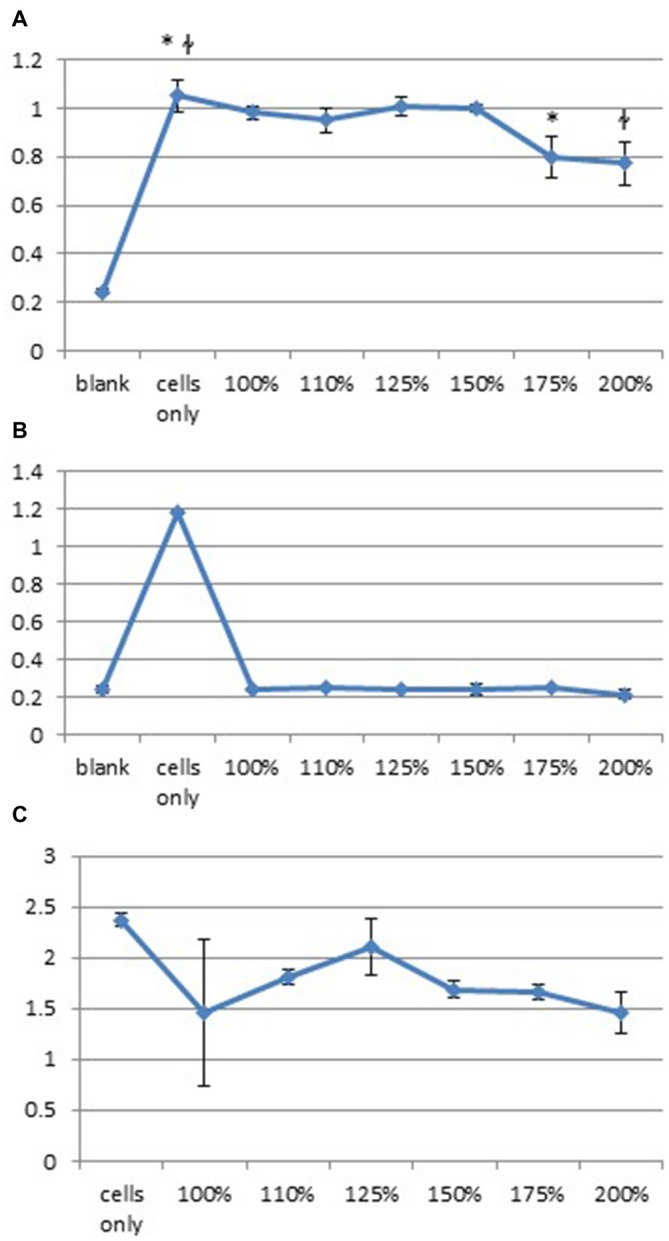
**High levels of glutamate decrease huDPSC proliferation.** Cell proliferation assays carried out with increased levels of glutamate from baseline levels show significantly decreased huDPSC proliferation 24 h after incubation with glutamate for **(A)** 15 min *p = 0.003, ^ł^*p* = 0.0023, **(B)** 24 h ANOVA *p* < 0.0001 and **(C)** Incubation for 15 min every hour for 6 h ANOVA *p* = 0.0031.

We hypothesized that DPSC loss may have resulted from glutaminergic toxicity resulting from TMS. An *in vitro* study of the effect of glutamate on DPSC was carried out using levels of glutamate calculated from data published by Zangen and Hyodo (2002) which showed upregulation of glutamate to levels 75% of normal physiological levels following TMS. This level was determined in our *in vitro* study we exposed DPSC to increasing levels of glutamate for 15 min, 24 h or 15 min/h for 6 h. All *in vitro* tests showed high levels of glutamate 75%-100% significantly decreased the number of DPSC seen at 24 h (one-way ANOVA; Figure 6). Overall we found that 24 h after the initial incubation, DPSC cell proliferation decreased or that no DPSC survived varied glutamate level exposure.

## Discussion

This exploratory study was designed to investigate the potential of TMS as a complementary therapy following the intracerebral transplantation of stem cells into the mammalian brain. We investigated whole brain TMS following human DPSC implantation in a rodent brain, rather than focal stimulation (localized to a specific brain area) to assess the global effect of TMS following the delivery of human DPSC. We found that significantly less transplanted DPSC survived in the rodent brains following five sessions of TMS stimulation at an intensity of 60% MSO over the course of 2 weeks (TMS_0.2 Hz_) compared with TMS_sham_ controls. Although the TMS_sham_ control used in this study is a clinically acceptable control in a human study, there may have been a low level of stimulation to the rodent brain as a result of the coil being orientated on a 90° angle, which may have provided a beneficial environment for implanted DPSC survival. Secondly, the surviving cells from the TMS_sham_ group predominantly remained in the injected cerebral hemisphere and did not migrate to the contralateral hemisphere. This is a somewhat surprising and unexpected finding. We have previously shown, that DPSC when transplanted into the rodent brain following stroke, migrated to the site of infarction, however, we also observed migration of DPSC to the contralateral non-stroke cerebral hemisphere (Leong et al., 2012). In the stroke model, stem cells are chemo-attracted to the site of the infarction by cytokines with one molecular pathway being CXCR4/CXCL-12 (SDF-1) signaling (Arthur et al., 2009). However, in this particular study there was no infarct, so the molecular mechanism whereby DPSC migrated throughout the rodent brain into both cerebral hemispheres is unclear. We can only hypothesize from our current experiments that our TMS_0.2 Hz_ paradigm does not allow for migration of implanted DPSC throughout the rodent brain.

Investigation of the differentiation phenotype of implanted DPSC demonstrated that implanted GFP-expressing DPSC treated with TMS predominantly expressed the neural progenitor cell marker nestin. This may suggest that TMS selected for a neural-type of progenitor cell within the DPSC population. In the sham controls we found neural antigen expressing GFP cells originally derived from implanted DPSC but were unable to find any such neural cells following TMS_sham_. Our immunohistochemical examination of implanted DPSC showed no neural markers (βIII-tubulin, GFAP or NeuN) were co-localized with the implanted DPSC in TMS_0.2 Hz_ treated animals.

As there were significantly less (50% decrease) implanted cells seen in the TMS_0.2 Hz_ treated brains we investigated the unexpected idea that TMS_0.2 Hz_ may have led to death of DPSC. This was investigated via Caspase-3 immunohistochemistry. We thus found a large proportion of surviving DPSC co-localized with the Caspase-3 apoptotic marker. To ensure that the endogenous neural stem cells (in the sub-ventricular zone, SVZ) were not affected in a similar manner by TMS_0.2 Hz_ treatment we investigated Caspase-3 expression by this stem cell population and did not find any co-localization at the sub ventricular zone in the rodent brain. Therefore TMS_0.2 Hz_ treatment appeared to be selectively detrimental to exogenously implanted stem cells and not the endogenous stem cell population. The finding that the majority of surviving DPSC expressed Caspase-3 may be explained as these cells were also destined to die and join the majority of their counterparts. While the study presented in this paper looked at Caspase-3 expression in the SVZ to ensure no endogenous neural stem cell was caused by TMS, a recent study by Abbasnia et al. (2015) showed following 1 week of rTMS at a low frequency (1 Hz) neural cells/progenitor cells derived from the SVZ of treated mouse brains, proliferation as measured by neurosphere formation was increased compared to cells derived from mice that received high frequency (30 Hz) rTMS for 1 week (Abbasnia et al., 2015).

We suggest a potential explanation for the detrimental effect of TMS_0.2 Hz_ treatment on DPSC survival may be due to increased glutamate levels in the brain parenchyma. It has previously been shown by Zangen and Hyodo (2002) that there is an up-regulation of glutamate during TMS and high levels of glutamate are cytotoxic. The study conducted by Zangen and Hyodo (2002) used a different TMS paradigm than that used in this study of 98% MSO at a frequency of 2 Hz, however, this is one of the only studies to our knowledge to measure glutamate levels in the rodent brain during TMS. Ideally, the current study would have benefited from conducting a similar study to that of Zangen and Hyodo (2002), however as it is an exploratory study, this was not able to be completed. An *in vitro* study completed, showed high levels of glutamate significantly decreased DPSC proliferation and survival. This may be the case *in vivo*.

Previous work carried out from this laboratory, in a rodent stroke model where the same population of human DPSC were injected 24 h post-stroke found only 2.3% of implanted DPSC survived at 4 weeks. It is known following ischemic stroke that there is an up-regulation of glutamate, which may support our suggestion that DPSC death may be due to increased levels of glutamate in the brain (Dirnagl et al., 1999; Leong et al., 2012).

There are a number of study limitations, which were a result of this study being the first of its kind to explore a complementary therapy of TMS and stem cell implantation. One such limitation was that we only investigated the effect of one set of TMS parameters. At the time of data collection there were few studies available to provide a useful basis for establishing the TMS intensity/stimulation frequency to achieve optimal outcomes. Thus, in this exploratory study we used a pragmatic approach and the highest frequency (0.2 Hz) and intensity (60% MSO) of TMS that was technically achievable for the stimulation period, without the coil overheating. It is likely, this intensity of stimulation resulted in significant activation of glutaminergic neurons with elevated glutamate levels and resultant toxicity to DPSC. It is possible that with lower stimulus intensities TMS may result in more favorable modulations in the cortical environment and less cell death of DPSC. Since data collection, there have been a number of other studies that have employed TMS in rodent models all with different protocols and research questions (Gersner et al., 2011; Wang et al., 2011; Tan et al., 2013), future experiments should focus on optimizing the TMS protocol (including optimizing the protocol design (continuous vis intermittent protocols), the intensity of the stimulation and the frequency of the stimulation for DPSC survival, based on the recently available evidence. Moreover, anesthetizing the animals during the TMS stimulation may have contributed to our results. Indeed Gersner et al. (2011); demonstrated an increase in hippocampal BDNF after 10 days of stimulation with similar intensity of stimulation in awake, but not anesthetized animals. Interestingly, a lower frequency of stimulation in their study resulted no increase of BDNF levels. Taking these findings together with our own, there may be a fine balance between providing an optimal environment for DPSC implantation by promoting increases in BDNF, without increasing glutamate, which may promote DPSC cell death. In future studies, we would suggest that measurement of glutamate levels in the brain during the TMS sessions via cannulation of the brain would provide a better understanding of the brain microenvironment and how this may affect implanted DPSC.

We hypothesized that TMS stimulation following injection of DPSC would provide an environment which would enhance the survival and neural differentiation of the implanted stem cells. Contrary to this hypothesis, our findings indicated that at the intensity and frequency tested (60% MSO, 0.2 Hz), TMS did not enhance transplanted DPSC survival and restricted migration of the implanted DPSC to the contralateral hemisphere. These data suggest that TMS may offer a novel complementary treatment to restrict stem cells to the site of implantation where they would be required for neuro-restoration following infarction. However, this restriction of movement throughout the brain parenchyma is at the expense of DPSC survival which is the ultimate goal, therefore, further studies are required to determine the optimal intensity of stimulation that can provide a brain environment which enhances the survival, differentiation and proliferation of implanted stem cells in the mammalian brain.

## Author Contributions

KLK: intracerebral brain injections, animal husbandry, collection of brain tissue, immunohistochemistry and analysis of results, *in vitro* experimentation, manuscript preparation and review. AES: transcranial magnetic stimulation of rats, analysis of results, manuscript preparation and review. LS: animal husbandry, immunohistochemistry, analysis of results. JI: immunohistochemistry, analysis of results. MCR and SAK: experimental concept, design and application for collaborative grant funding, analysis of results, manuscript preparation.

## Funding

This project was funded by a Robinson Research Institute collaborative research grant, The University of Adelaide. AES is supported by an NHMRC-ARC dementia training fellowship (ID 1097397).

## Conflict of Interest Statement

The authors declare that the research was conducted in the absence of any commercial or financial relationships that could be construed as a potential conflict of interest. The reviewer SIH and handling Editor declared their shared affiliation, and the handling Editor states that the process nevertheless met the standards of a fair and objective review.

## Footnotes

^1^www.rockland-inc.com

^2^www.abcam.com

^3^www.cellsignal.com

^4^www.invitrogen.com
